# Chronic Cigarette Smoking Impairs Erectile Function through Increased Oxidative Stress and Apoptosis, Decreased nNOS, Endothelial and Smooth Muscle Contents in a Rat Model

**DOI:** 10.1371/journal.pone.0140728

**Published:** 2015-10-22

**Authors:** Yun-Ching Huang, Chih-Chien Chin, Chih-Shou Chen, Alan. W. Shindel, Dong-Ru Ho, Ching-Shwun Lin, Chung-Sheng Shi

**Affiliations:** 1 Division of Urology, Department of Surgery, Chang Gung Memorial Hospital, Chiayi, Taiwan; 2 Graduate Institute of Clinical Medical Sciences, College of Medicine, Chang Gung University, Taoyuan, Taiwan; 3 Division of Colon and Rectal Surgery, Department of Surgery, Chang Gung Memorial Hospital, Chiayi, Taiwan; 4 Department of Urology, University of California Davis, Davis, CA, United States of America; 5 Knuppe Molecular Urology Laboratory, Department of Urology, University of California San Francisco, San Francisco, CA, United States of America; Max-Delbrück Center for Molecular Medicine (MDC), GERMANY

## Abstract

Cigarette use is an independent risk factor for the development of erectile dysfunction (ED). While the association between chronic smoking and ED is well established, the fundamental mechanism(s) of cigarette-related ED are incompletely understood, partly due to no reliable animal model of smoking-induced ED. The present study was designed to validate an *in vivo* rat model of chronic cigarette-induced ED. Forty 12-week old male Sprague-Dawley rats were divided into 4 groups. Ten rats served as control group and were exposed only to room air. The remaining 30 rats were passively exposed to cigarette smoke (CS) for 4 weeks (n = 10), 12 weeks (n = 10), and 24 weeks (n = 10). At the 24-week time point all rats were assessed with intracavernous pressure (ICP) during cavernous nerve electrostimulation. Blood and urine were collected to measure serum testosterone and oxidative stress, respectively. Corporal tissue was assessed by Western blot for neuronal nitric oxide synthase (nNOS). Penile tissues were subjected to immunohistochemistry for endothelial, smooth muscle, and apoptotic content. Mean arterial pressure (MAP) was significantly higher in 24-week cigarette exposed animals compared to the control animals. Mean ICP/MAP ratio and cavernosal smooth muscle/endothelial contents were significantly lower in the 12- and 24-week rats compared to control animals. Oxidative stress was significantly higher in the 24-week cigarette exposed group compared to control animals. Mean nNOS expression was significantly lower, and apoptotic index significantly higher, in CS-exposed animals compared to control animals. These findings indicate that the rat model exposure to CS increases apoptosis and oxidative stress and decreases nNOS, endothelial and smooth muscle contents, and ICP in a dose dependent fashion. The rat model is a useful tool for further study of the molecular and cellular mechanisms of CS-related ED.

## Introduction

Erectile dysfunction (ED) currently affects 52% men between subject ages 40 and 70 years in United States; the prevalence of this condition increases with age [1]. Although ED is not life-threatening, it is associated with substantially negative impact on the physical and psychosocial health of men and their partners [2].

Cigarette smoke (CS) is an independent risk factor for the development of ED [3]. In the Massachusetts Male Aging Study, CS almost doubled the odds of developing moderate or complete ED at up to 10 years of follow-up in men aged 40–70 years at baseline [4]. While the deleterious effects of CS on ED are well established, the pathophysiological mechanisms of ED in tobacco users remain poorly understood [5].

Animal models for the study of CS-related ED have been established. In a dog model, Juenemann et al. demonstrated that acute exposure to CS caused impairment in penile arterial inflow and veno-occlusion with lack of complete erection during cavernous nerve electrostimulation [6]. Bivalacqua et al. reported that mice exposed to CS for 5 hours per day, 5 days per week for 3 weeks impaired erectile function. This was thought to be related to increased penile reactive oxygen species (ROS) signaling and inducible nitric oxide synthase activity [7]. Imamura reported similar results in rabbit cavernosal tissue *in vitro* [8].

While rats are the most widely contemporary animal model for ED, there have been relatively few studies using rats for the study of CS related ED [9–11]. Xie et al. demonstrated that passive smoking for 1 hour per day, 5 days per week for 8 weeks in rats led to hypertension and decreased penile neuronal nitric oxide synthase (nNOS) content compared to controls; interestingly, in this study cavernous erectile response to electrostimulation was not significantly diminished in tobacco exposed animals compared to controls [10]. Park et al. reported that acute and chronic exposure to CS in a rat was associated with hypertension, decreased testosterone levels and penile smooth muscle content, and declined in penile hemodynamic response to electrostimulation of cavernous nerve [11]. Further characterization of the tissue and hemodynamic effects of CS in a widely available rat strain would greatly facilitate the understanding of tobacco related ED.

The present study was designed to validate an *in vivo* rat model of chronic CS-induced ED. We hypothesized that exposure to tobacco smoke would have a dose-dependent, negative effect on penile hemodynamic parameters which would be associated with pathological changes of the penile tissues.

## Materials and Methods

### Animal Groups and Experimental Design

Forty 12-week old male Sprague-Dawley rats were obtained from BioLASCO Taiwan Co., Ltd. The cares, treatments, and procedures of rats were in accordance with the rules of Association for Assessment and Accreditation of Laboratory Animal Care International and approved by the Institutional Animal Care and Use Committee at our Laboratory Animal Center, Department of Medical Research, Chang Gung Memorial Hospital at Chiayi (2012121822).

The rats were divided into 4 groups. Ten air-exposed rats served as non-smoking controls (control group) and all underwent erectile function testing at the age of 36 weeks. The remaining, age-matched 30 rats were passively exposed to CS for 4 weeks (4-week group, initially exposed at 32 weeks of age), 12 weeks (12-week group, initially exposed at 24 weeks of age) and 24 weeks (24-week group, initially exposed at 12 weeks of age)). After the last exposure to CS, all rats were placed in a metabolic cage for 24-hour urine collection. Urine samples were obtained to measure systemic oxidative stress. While in the metabolic cage, all rats had free access to water and food [12]. After urine collection, all 36-week old rats also underwent erectile function testing. Animals were then sacrificed. Serum samples were obtained to measure serum testosterone. Penile tissues were collected for Masson’s trichrome, immunohistochemical and Western blot analyses.

### Cigarette Smoke exposure

**Fig 1:**
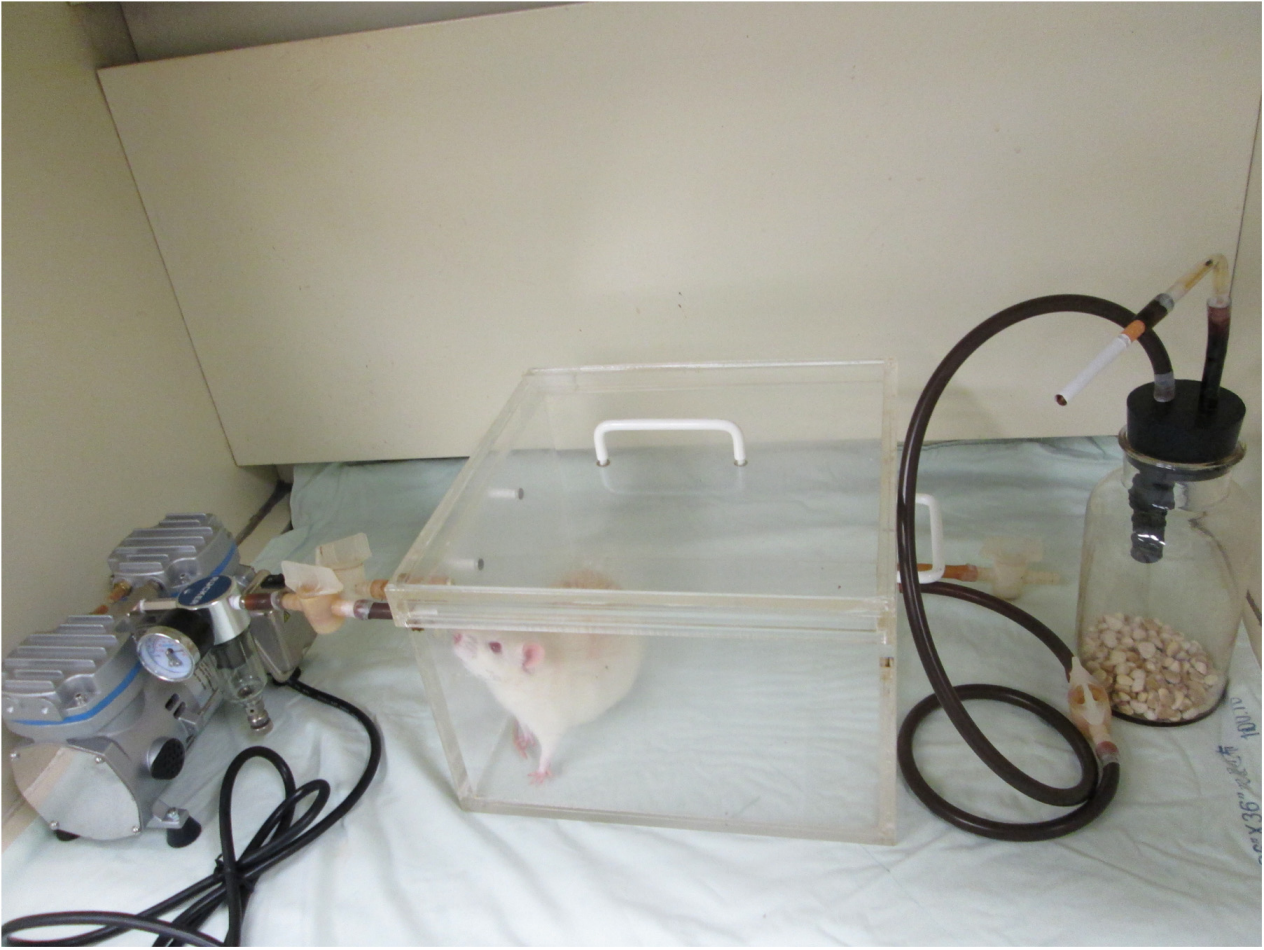
Smoke exposure equipment. The rat was housed in an enclosed plastic cage (28x28x19 cm) and subjected to a constant influx of cigarette smoke using a small air pump. One lighted cigarette was placed into an inverted tube connected to a peristaltic pump communicating with the cage.

All rats were housed in enclosed plastic cages measuring 28×28×19 cm. Rats exposed to CS were housed in cages treated with a constant influx of CS using a small air pump. One lighted cigarette was placed into an inverted tube connected to a peristaltic pump communicating with the cage. The smoke was distributed by a small fan and exited through a second opening (Fig 1) [13]. One cigarette (contents: 10 mg of tar and 0.8 mg of nicotine, Marlboro, Philip Morris, Richmond, VA, USA) with filter was combusted consecutively to exhaustion at a rate of 10 cigarettes per hour. The rats were exposed to 2 hours of CS per day for 5 days per week.

### Erectile Function Testing

All rats were anesthetized with Zoletil 50 (20 mg/kg) via intra-muscular injection. A lower midline abdominal incision was made and the cavernous nerves were isolated bilaterally. The penis was denuded of overlying skin and cannulated with a heparinized 23-gauge needle connected to a real-time continuous pressure transducer. The cavernous nerves were then stimulated with a stainless steel bipolar hook electrode attached to a multi-jointed clamp; stimulation parameters were 50 seconds continuous trains at 20 Hz, and 1.5 mAmp (A-M Systems, Sequim, WA, USA). Real-time response of the erectile tissue was determined by a change in intracavernous pressure (ICP) using LabView 6.0 software (National Instruments, Austin, TX, USA)[14]. The maximum change in ICP was utilized for further analysis. After functional testing, systemic blood pressure was measured via aortic cannulation. Mean arterial pressure (MAP) was calculated by the formula MAP = (2/3 diastolic blood pressure + 1/3 systolic blood pressure). After aortic puncture, serum and urine samples were obtained, and then animals were sacrificed with bilateral thoracotomy [15].

### Oxidative Stress Assay

We determined the level of malondialdehyde (MDA, the most commonly used marker for lipid peroxidation) and 8-hydrox-2’-deoxyguanosine (8-OHdG, one of the predominant forms of free radical-induced oxidative DNA damage products) in urine [16, 17].

To analyze MDA and 8-OHdG level, the urine was centrifuged at 3000 g for 5 min at 4°C. The concentration of MDA and 8-OHdG were measured using a colorimetric 2-thiobarbituric acid reactive substances microplate assay kit (Oxford Biomedical Research, Rochester Hills, MN, USA) and an 8-OHdG Enzyme-Linked Immunosorbent Assay kit (Trevigen, Gaithersburg, MD, USA), respectively, according to the manufacturer ‘s instructions [18]. The urinary concentration of MDA and 8-OHdG were normalized to urinary creatinine to avoid the effect of urine volume fluctuation [18].

### Western Blot Analysis

After euthanasia, the urethra was dissected free from penile tissue samples. About 50 mg of the rat penis tissue was homogenized in 1 ml tissue protein extraction exigent (Thermo Fisher Scientific Inc., Waltham, MA, USA). The lysate was centrifuged at 140,000 rpm for 15 minutes, and the supernatant was collected as the protein samples.

Tissue lysates containing 40 g of protein were electrophoresed in gradient SDS-PAGE and then transferred onto a PVDF membrane (Millipore, Darmstadt, Germany). Detection of protein on the membrane was performed with the ECL kit (Amersham Life Sciences, Pittsburgh, PA, USA) using a rabbit anti-nNOS (Santa Cruz Biotechnology, Dallas, TX, USA). Before re-probing with an anti—actin antibody, the membrane was stripped in 62.5mM Tris-HCl, pH 6.7, 2% SDS, 10 mM 2-mercaptoethanol at 56°C for 30 minutes and then washed four times in 1xTBST. Results were quantified by densitometry.

### Immunohistochemical Staining

After euthanasia, tissue samples were fixed in cold 2% formaldehyde and 0.002% picric acid in 0.1 M phosphate buffer, pH 8.0, for 4 hours followed by overnight immersion in buffer containing 30% sucrose. The specimens were then embedded in OCT Compound (American Master Tech Scientific, Lodi, CA, USA) and stored at -70°C until use. Sections were cut at 5 μm, mounted into charged slides and air dried for 5 minutes. Representative slides were stained with Masson’s trichrome for connective tissue and smooth muscle histology.

For immunohistochemical examination, tissue sections were stained with mouse anti-rat endothelial cell antigen-1 (RECA-1, Abcam Inc, Cambridge, MA, USA) and terminal deoxynucleotidyl transferasemediated deoxyuridine triphosphate nick-end labeling (TUNEL, Roche Diagnostics Corporation, Indianapolis, IN, USA) using standard techniques [15].

### Image and Statistical Analysis

Image analysis was performed by computerized densitometry using Image-Pro plus imaging software (Media Cybernetics, Silver Spring, MD, USA) coupled to a digital camera (Nikon DXM1200) and ACT-1 software (Nikon Instruments Inc., Melville, NY, USA).

To quantify Masson’s trichrome staining, one fields of the penis comprising one-half of the corpus cavernosum but excluding the sinusoidal spaces were analyzed for smooth muscle (stained in red) and collagen (stained in blue) at 40 x magnification. Data were expressed as the smooth muscle/collagen ratio. For RECA-1 staining, one fields of the penis comprising one-half of the corpus cavernosum were analyzed at 40 x magnification and results were expressed as the percentage of positive area vs. total area of the corpus cavernosum. For TUNEL staining, five randomly selected fields of corpus cavernosum were examined at x100 magnification; results were expressed as the number of TUNEL-positive nuclei in the corpus cavernosum. All the quantities of Immunohistochemical staining were done in a blinded fashion by two reviewers.

Data were analyzed with Prism version 6 (GraphPad Software, La Jolla, CA, USA) and expressed as mean ± standard error of the mean for all continuous variables. Continuous data were analyzed using one-way analysis of variance. The Tukey–Kramer test was used for post hoc comparisons. Statistical significance was set at *P* < 0.05.

## Results

### Body Weight and Functional Study

Although there was no statistically significant difference in total body weight, there was a trend towards decreased body weight in the rats that exposed to CS (Table 1).

**Table 1:** Total body weight and erectile response during cavernous nerve electrostimulation.

		Cigarette smoke exposure			
	Control	4-week	12-week	24-week	*p* value
Body weight (gram)	722 ± 17.0	693 ± 15.7	653 ± 17.7	667 ± 29.7	0.0847
Peak ICP(cm-H_2_O)	101.2±9.76	86.7±15.79	75.4±8.93	74.3±14.99	0.4116
MAP (cm-H_2_O)	123.5±8.06	139.4±11.42	158.7±11.27	166.6±7.11^*^	0.0190
ICP/MAP ratios	0.87±0.125	0.56±0.096	0.50±0.068^*^	0.44±0.085^*^	0.0189

ICP = intracavernous pressure

MAP = mean arterial pressure.

* Versus control group *p* < 0.05.

Mean ICP/MAP ratio was significantly lower in the 12-week and 24-week groups compared to the control group. The 4-week group also had lower mean ICP/MAP ratio than the control group but the difference did not reach statistical significance (*p* = 0.120). Average MAP was significantly higher in the 24-week group compared to the control group (*p* = 0.027). The 4-week and 12-week group also had higher average MAP than the control group but the difference did not reach statistical significance. Interestingly, peak ICP was not significantly different between the four groups (Table 1).

### Testosterone and Oxidative Stress State

There were no significant differences in mean testosterone level between the four groups (Table 2).

**Table 2:** Plasma testosterone concentration and urine oxidative stress state.

		Cigarette smoke exposure			
	Control	4-week	12-week	24-week	*p* value
Testosterone (ng/mL)	4.3±0.67	2.8±0.63	3.2±0.40	3.0±0.72	0.2882
MDA/Creatinine (μmol/g)	10.5±1.71^*^	10.2±1.62^*^	10.7±1.17^†^	21.2±3.59	0.0035
8-OHdG/Creatinine (ng/mg)	35.1±2.30^*^	51.2±6.44	56.6±7.57	79.8±15.65	0.0069

MDA = malondialdehyde

8-OHdG = 8-hydrox-2’-deoxyguanosine.

The urinary concentration of MDA and 8-OHdG were normalized to urinary creatinine to avoid the effect of urine volume fluctuation.

* Versus 24-week group *p* < 0.01.

† Versus 24-week group *p* < 0.05.

MDA level in the 24-week group was significantly higher relative to all other groups; there was no significant difference in MDA between control, 4-week and 12-week groups. The level of 8-OHdG in the 24-week group was significantly higher than what was observed in control group; no other significant differences between groups were noted for 8-OHdG although there was a trend towards higher levels with greater exposure (Table 2).

### nNOS Western Blot

**Fig 2:**
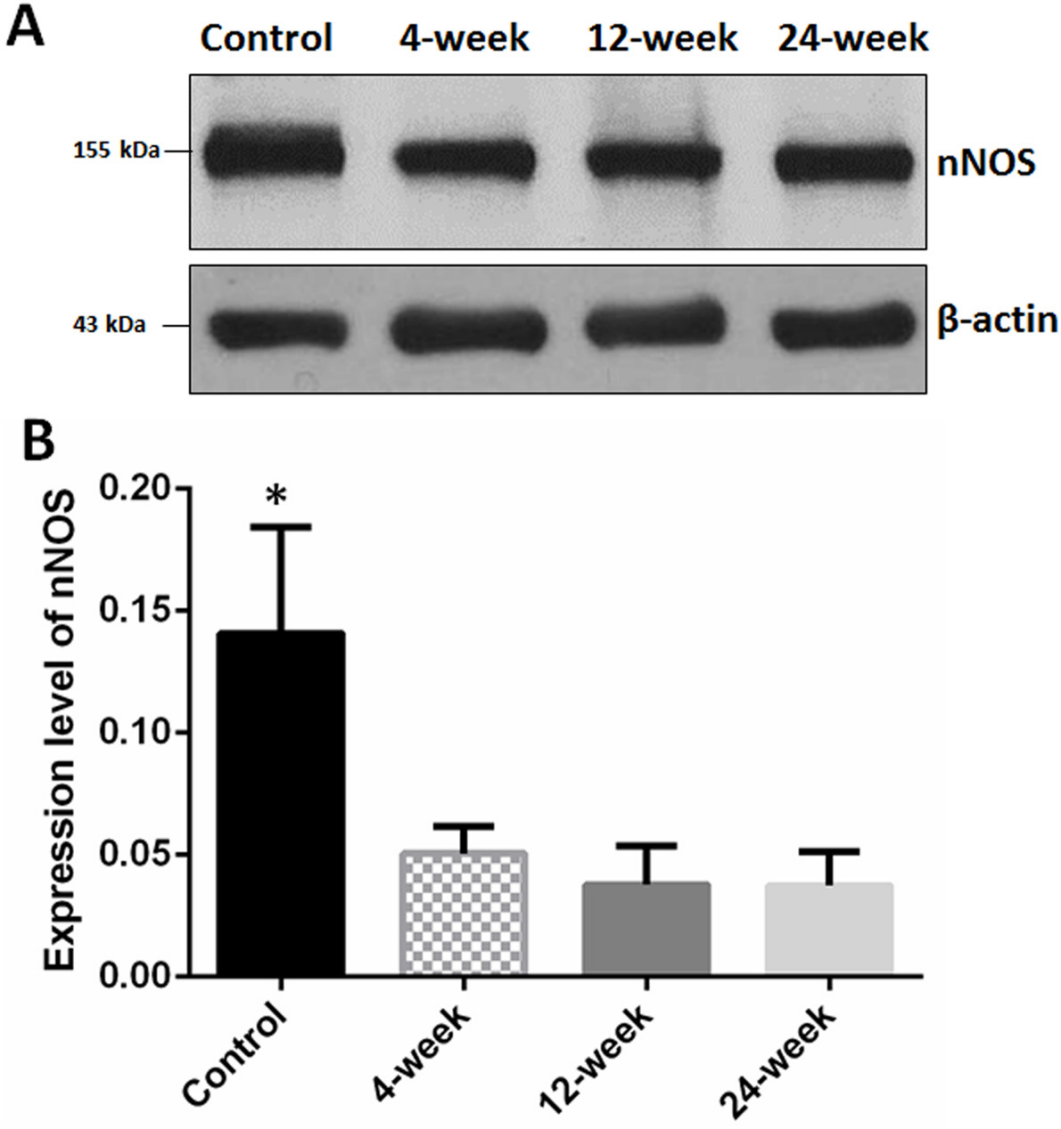
Western blot for neuronal nitric oxide synthase (nNOS). (A) Western blot normalized to β-actin; (B) mean nNOS positivity. Mean nNOS was significantly higher in the control group compared with all others (* versus all other groups *p* < 0.05).

Protein expression of penile nNOS was significantly higher in the control group (0.14±0.044) compared with 4-week (0.05±0.035), 12-week (0.038±0.0052) and 24-week (0.037±0.0053) groups (*p* = 0.012). There was no significant difference between smoking groups. Representative images of nNOS Western blot are presented in Fig 2.

### Endothelial Integrity

**Fig 3:**
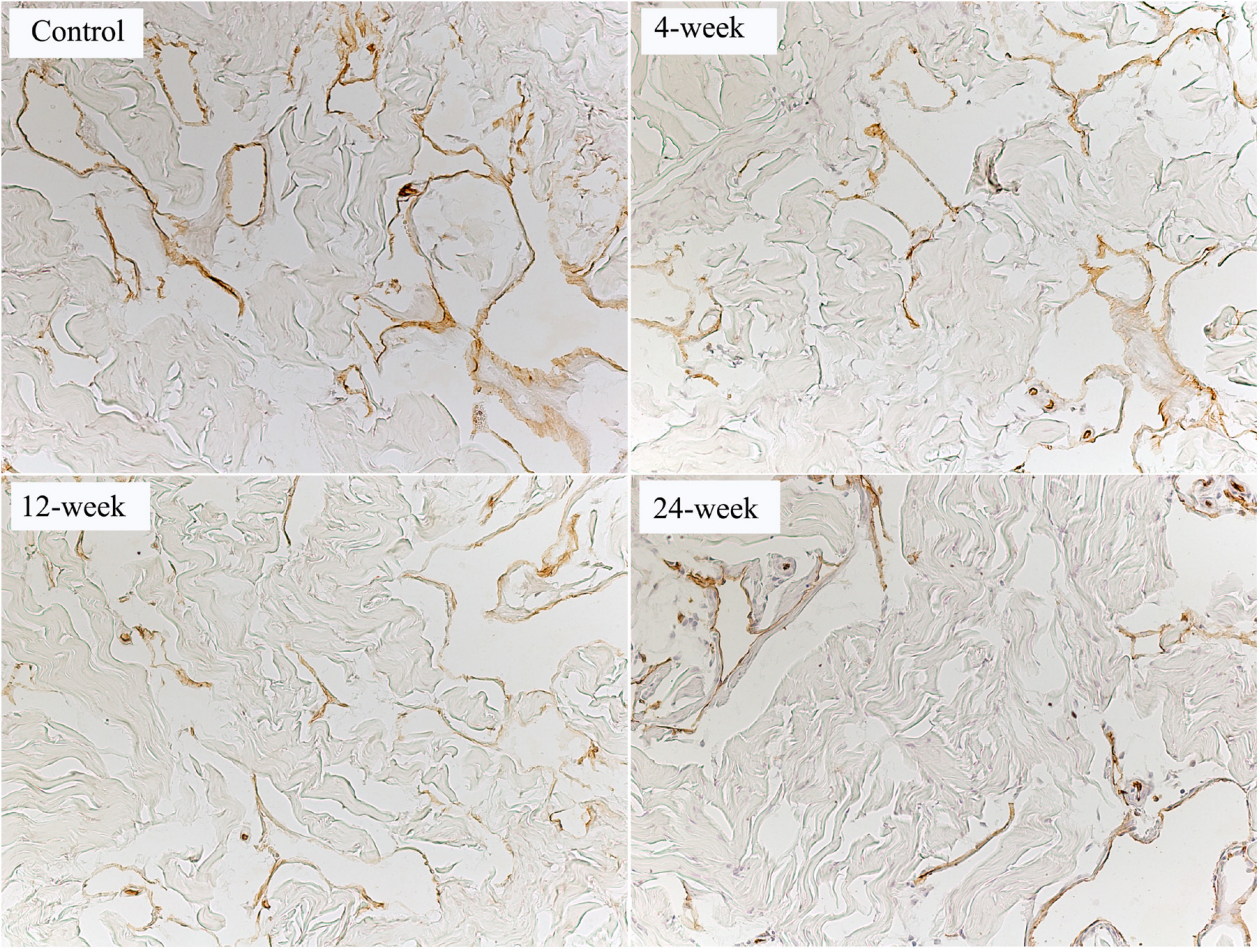
Endothelium content in the corpus cavernosum. Representative images from rats in the control, 4-week, 12-week and 24-week groups. Endothelial cells are stained brown with the rat endothelial cell antigen-1 (RECA-1) antibody. The intense positivity of RECA-1 was higher in control than in 12-week and 24-week groups. Magnification is x200.

RECA-1 staining for endothelial tissue in the corpus cavernosum was significantly higher in the control group relative to the 12-week and 24-week groups. There was no significant difference in positivity between smoking groups (Table 3). Representative images of RECA-1 staining are presented in Fig 3.

**Table 3:** RECA-1, Masson’s trichrome and TUNEL stain data in the experimental animals.

		Cigarette smoke exposure			
	Control	4-week	12-week	24-week	*p* value
RECA-1 percentage (%)	5.1±0.91	3.6±0.55	2.5±0.41^*^	2.1±0.30^*^	0.0099
SM/collagen ratio (%)	21.7±2.94	17.9±3.08	8.5±1.14^†^	9.2±1.66^*^	0.0013
TUNEL (number)	5.7±1.16	9.8±1.71^‡^	29.2±9.05^*^	36.3±8.21^†^	0.0013

RECA-1 = rat endothelial cell antigen-1

SM = smooth muscle

TUNEL = terminal deoxynucleotidyl transferase dUTP nick-end labeling.

* Versus control group *p* < 0.05.

† Versus control group *p* < 0.01.

‡ Versus 24-week group *p* < 0.05.

### Smooth Muscle Content

**Fig 4:**
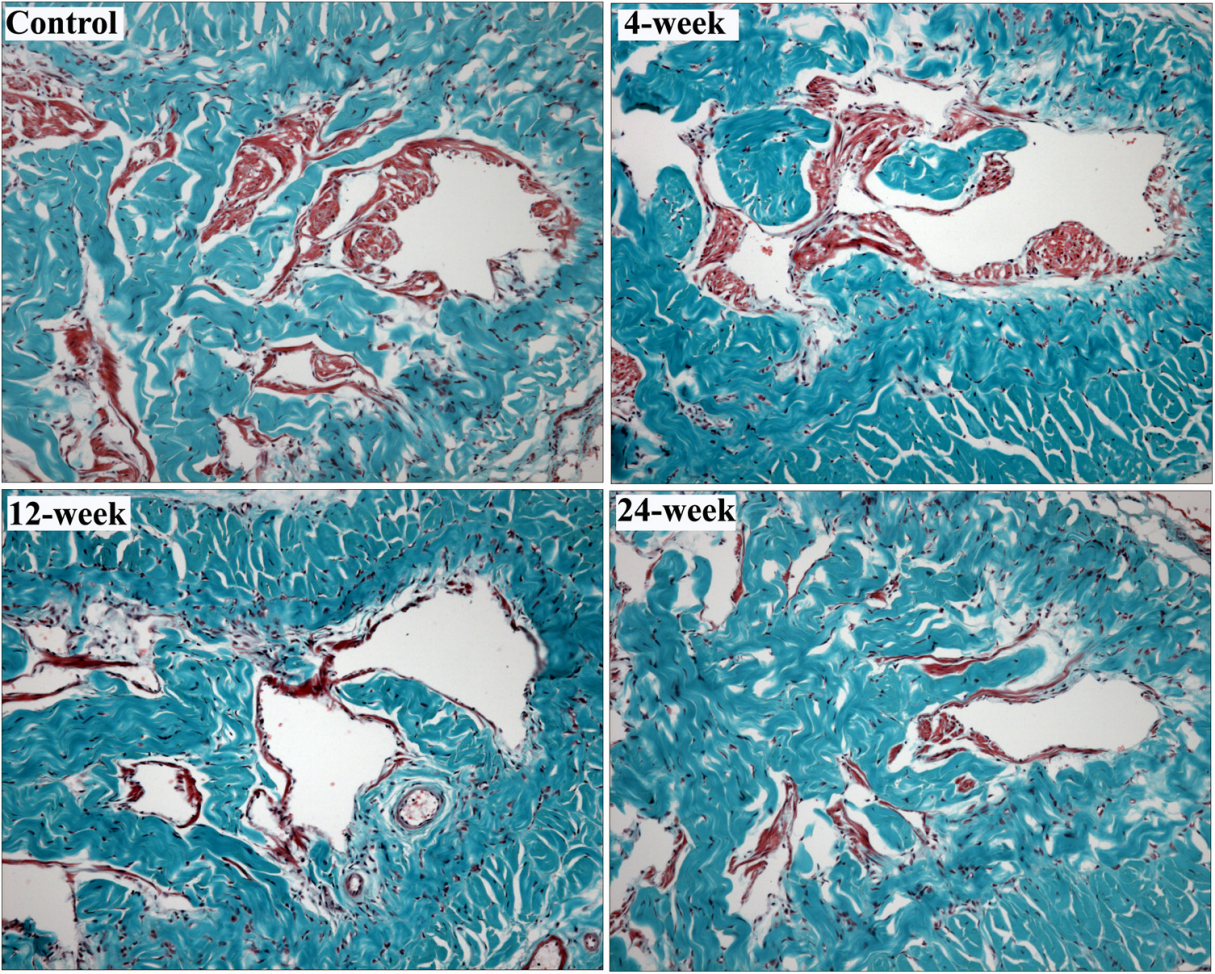
Smooth muscle content in the corpus cavernosum. Representative images from rats in the control, 4-week, 12-week and 24-week groups were shown. Smooth muscle and connective tissue are stained with red and blue, respectively, using the trichrome method. The smooth muscle content was higher in control group than in 12-week and 24-week groups. Magnification is x100.

Masson’s trichrome demonstrated significantly higher smooth muscle to collagen ration in control animals compared to the 12-week and 24-week groups (Table 3). There was no significant difference in smooth muscle to collagen ration between smoking groups. Representative images of Masson’s trichrome staining are presented in Fig 4.

### TUNEL Immunohistochemistry

**Fig 5:**
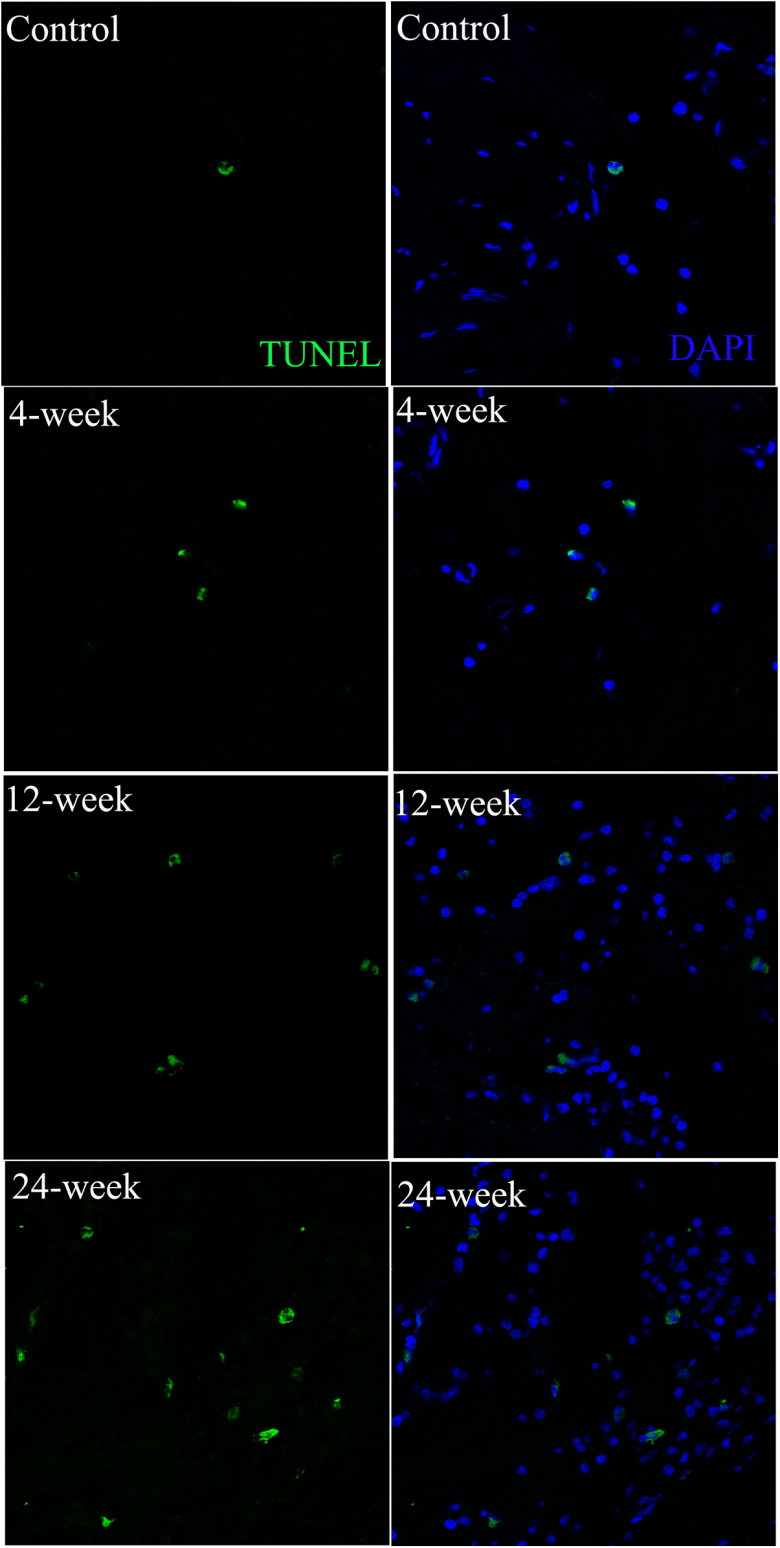
Analysis of apoptosis-positive cells by TUNEL stain. The corpus cavernosum was stained with TUNEL stain (green) and DAPI (blue) for the visualization of apoptotic cells and cell nuclei, respectively. The result showed the increase of the number of apoptosis-positive cells from control to 4-week, to 12-week and to 24-week. Magnification is x800.

TUNEL-positive cells were significantly less frequent in control animals compared to the 12-week and 24-week groups (Table 3). The number of TUNEL positive cells was significantly higher in the 24-week group than in the 4-week group (*p* = 0.016). Representative images of TUNEL staining are presented in Fig 5.

## Discussion

This work represents an expansion of our knowledge based on the rat model system for investigation of tobacco related erectile impairment [6, 7]. The rat model offers several advantages. First and foremost, the cavernous nerve in rat can be more easily identified and stimulated to assess erectile response than in mouse. Rats are inexpensive, easy to house and take care, less likely to develop anesthesia related complications, and have been widely used in contemporary studies of penile hemodynamics than dog [9, 19].

The major goal of this study was to determine the time-dependent effects of CS exposure on the physiological and systemic parameters of rats in relation to ED processes. To our best knowledge, the results of the current study that a rat model of CS-induced ED is associated with decreased penile nNOS content, diminished endothelial and muscular content, increased penile apoptosis, and increased systemic oxidative stress is first time to be demonstrated. We observed that 12 and 24 weeks of CS exposure in rats resulted in reduced ICP, increased MAP, and decreased ICP/MAP ratios. The decline in ICP/MAP ratio in rats subjected to prolonged CS exposure is consistent with the human condition of tobacco-related ED.

Several epidemiological studies have clearly documented that tobacco is a dose dependent risk factor for hypertension [20]. In a mouse model, CS exposure for 16 weeks showed a trend towards increased blood pressure; this trend was statistically significant compared to non-exposed mice at 32 weeks of CS exposure [21]. These results are similar to ours. Although the mechanism of elevated blood pressure in our model was not investigated, it has been reported that the increase in blood pressure with CS is mediated via the stimulation of the sympathetic nervous system, impairment of endothelial function, and increased arterial stiffness [22, 23].

Long-term smoking has been shown to diminish the capacity of endothelium to counterbalance external factors that cause endothelial dysfunction in humans [24]. In our animal model we found that greater CS exposure was associated with poorer endothelial staining and declines in smooth muscle to collagen ratio.

Previous investigations using animal models of tobacco exposure for the study of ED demonstrated increased risk of hypertension but no significant difference in ICP/MAP ratio [10]. The difference in hemodynamic outcomes between the study of Xie et al. and our series may be a result of the duration of CS exposure (40 in the prior study vs 120 hours in the long term exposure arm of our study). In our study protocol, ICP showed a trend to decrease at smoking groups, and MAP was statistically significant difference from that of control rats at 24 weeks of CS exposure. We postulate that with increasing CS exposure, the hypertensive and other negative tissue effects accumulate to produce declines in ICP/MAP ratio.

Although the exact pathophysiology of CS-induced ED has not been determined, the association between CS and oxidative stress has been observed in mice and humans and there is a consensus that CS increases ROS signaling and alternation in NOS activities [7, 25]. Free radicals in tobacco users could be generated from the CS itself; circulating or in situ activated neutrophils, monocytes, and macrophages; and endogenous sources of ROS, such as uncoupled endothelial NO synthase, xanthine oxidase, and the mitochondrial electron transport chain [26, 27]. In our study long term CS-exposed animals had higher levels of oxidative stress (as determined by MDA and 8-OHdG expression in urine) when comparing to control animals. We speculate that oxidative stress markers in urine could serve as a marker for ED in men who smoke. The validity and clinical application of these markers for oxidative stress in ED requires additional study.

The principal limitations of our study include the limited number of animals, and different age of CS exposure with a lack of their own control in 4-week and 12-week groups. The voltage-dose response is not available in the erectile function testing. The tobacco exposure was of high intensity but limited and intermittent duration; this differs from the typical human exposure which occurs intermittently throughout the course of a day. Whether or not this difference in exposure pattern is relevant remains to be determined, and our findings provide convincing insights into a rat model of CS-induced ED. Remaining questions are about the influence of stress to the rats during exposure to CS. These important questions will be the subjects of additional research projects.

## Conclusions

In this pilot study we found that chronic CS exposure increases systemic oxidative stress state as indicated by alternations in the damage markers of MDA and 8-OHdG in urine. Long-term CS exposure was also associated with declines in the smooth muscle, endothelial, and neuronal components, which is important for penile erection. Further studies to verify the molecular mechanisms underlying the relationship between CS and ED are warranted.
